# Optimal Ablation Settings Predicting Durable Scar Detected Using LGE-MRI after Modified Left Atrial Anterior Line Ablation

**DOI:** 10.3390/jcm11030830

**Published:** 2022-02-04

**Authors:** Mathias Forkmann, Christian Mahnkopf, Marcel Mitlacher, Marc Wolff, Beatriz Tose Costa Paiva, Sonia Busch

**Affiliations:** 1 Klinikum Coburg, Department of Cardiology and Angiology, 96450 Coburg, Germany; christian.mahnkopf@regiomed-kliniken.de (C.M.); marcel.mitlacher@regiomed-kliniken.de (M.M.); biatose@gmail.com (B.T.C.P.); sonia.busch@regiomed-kliniken.de (S.B.); 2 Johnson & Johnson Medical GmbH, Biosense Webster, Robert-Koch-Str.1, 22851 Norderstedt, Germany; mwolff01@its.jnj.com

**Keywords:** cardiac arrhythmias, atrial fibrillation, catheter ablation

## Abstract

(1) Background: The modified anterior line (MAL) has been described as an alternative to the mitral isthmus line. Despite better ablation results, achieving a bidirectional line block can be challenging. We aimed to investigate the ablation parameters that determine a persistent scar on late-gadolinium enhancement magnet resonance imaging (LGE-MRI) as a surrogate parameter for successful ablation 3 months after MAL ablation. (2) Methods: Twenty-four consecutive patients who underwent a MAL ablation have been included. The indication for MAL was perimitral flutter (n = 5) or substrate ablation in the diffuse anterior left atrial (LA) low-voltage area in persistent atrial fibrillation (AF) (n = 19). The MAL was divided into three segments: segment 1 (S1) from mitral annulus to height of lower region of left atrial appendage (LAA) antrum; segment 2 (S2) height of lower region of LAA antrum to end of upper LAA antrum; segment 3 (S3) from end of upper LAA antrum to left superior pulmonary vein. Ablation was performed using a contact force irrigated catheter with a power of 40 Watt and guided by automated lesion tagging and the Ablation Index (AI). The AI target was left to the operator’s choice. An inter-lesion distance of ≤6 mm was recommended. The bidirectional block was systematically evaluated using stimulation maneuvers at the end of procedure. All patients underwent LGE-MRI imaging at 3 months, regardless of symptoms, to identify myocardial lesions (scars). (3) Results: Bidirectional MAL block was achieved in all patients. LGE-MRI imaging revealed scarring in 45 of 72 (63%) segments. In all three segments of MAL, ablation time and AI were significantly higher in scarred areas compared with non-scar areas. The mean AI value to detect a durable scar was 514.2 in S1, 486.7 in S2 and 485.9 in S3. The mean ablation time to detect a scar was 20.4 s in S1, 22.1 s in S2 and 20.2 s in S3. Mean contact force and impedance drop were not significantly different between scar and non-scar areas. (4) Conclusions: Targeting optimal AI values is crucial to determine persistent left atrial scars on an LGE-MRI scan 3 months after ablation. AI guided linear left atrial ablation seems to be effective in producing durable lesions.

## 1. Introduction

Perimitral flutter is a common macro-re-entrant arrhythmia during and after left atrial ablation procedures in patients with atrial fibrillation (AF) [1,2]. In addition, a linear ablation approach has been proposed for substrate modification in patients with diffuse low-voltage-areas [3]. The modified anterior line (MAL), deployed between the anterolateral mitral annulus (MA) and the left superior pulmonary vein (LSPV), has been described as an alternative to the lateral mitral isthmus line [4]. Radiofrequency (RF) is the dominant energy source used for catheter ablation outside the pulmonary veins (PV) [5]. Several factors determine the size and depth of RF lesions: power, impedance, temperature, duration and contact force (CF) [6,7]. Ablation parameters such as CF, ablation time, catheter stability and impedance drop can be monitored simultaneously by electroanatomical mapping systems. The Ablation Index (AI) (CARTO 3^®^ V6, Biosense Webster, Inc., Diamond Bar, CA, USA) incorporates CF, time and power in a weighted formula, and has been introduced as a marker for ablation lesion quality.

Late gadolinium enhancement magnetic resonance imaging (LGE-MRI) has become increasingly evident in cardiac electrophysiology and can play a role in identifying ablation lesions and localizing ablation [8,9,10]. A previous study has reported ablation time and Force-Time Integral (FTI) values as important parameters for the determination of permanent atrial scarring in LGE-MRI [11]. However, as FTI is limited by the exclusion of power delivery, AI seems to be a more accurate parameter for predicting ablation lesion. The aim of this study was to identify the optimal ablation parameters that result in durable left atrial scarring during LGE-MRI after deploying a MAL.

## 2. Methods

### 2.1. Patient Population

This observational study comprised 24 consecutive patients who underwent RF catheter ablation from March 2018 to September 2019. Patients were included if a MAL was deployed during the procedure. The indication for a MAL was perimitral flutter in 5 cases and substrate ablation for diffuse anterior low-voltage areas in persistent atrial fibrillation in 19 patients. Baseline characteristics are shown in Table 1.

### 2.2. Ablation Procedure

**Figure 1:**
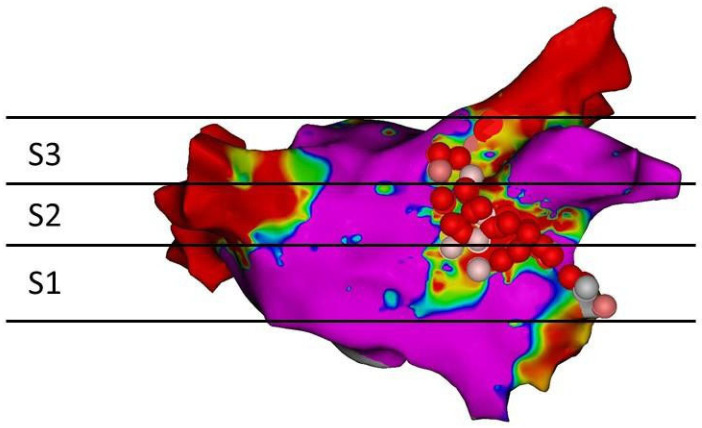
Three segments of a MAL. Voltage map of the left atrium. Violet areas reflect normal voltage (>0.5 mV), red areas a voltage <0.1 mV. VisiTags colour-coded by AI value. S1, segment 1; S2, segment 2; S3 segment 3.

All patients signed a written informed consent form. A transoesophageal echocardiography was performed before the procedure to rule out left atrial thrombus formation. The procedure was carried out in a fasting state under analgo-sedation (fentanyl sodium and propofol), and heparin was injected to maintain an activated clotting time of >300 s. Ablation was supported by the CARTO3^®^ V6 system. First, the left and right pulmonary veins (PV) were isolated. After, a MAL was deployed as previously described (4). The anterolateral mitral annulus and the ostium of the left superior PV were then connected (Figure 1).

Irrigated RF energy was delivered using the CF-sensing THERMOCOOL SMARTTOUCH^®^ SF catheter with a default power setting of 40 W and an irrigation of 17 mL/min. The ablation catheter was introduced in a steerable sheath (Agilis^®^, Abbott). The line was performed with counter clock rotation and progressive release of the sheath curve. Ablation lesions were deployed in a point-by-point setting guided by AI. The operator designated the AI target. Automated lesion tagging (CARTO VISITAG Module™, Biosense Webster, Inc., Diamond Bar, CA, USA) was used to mark the location of each lesion. The AI threshold for VisiTags to turn red was 500. An inter-lesion distance of ≤6 mm was recommended. The following VisiTag settings were used: catheter position stability: minimum time 3 s, maximum range 2.5 mm; force over time: time 25%, minimum force 3 g; lesion tag size: 2 mm. It was left to the operator to stop an ablation or not; however, most ablations were continued until local EGM elimination.

Bidirectional block was confirmed in sinus rhythm using the aforementioned differential pacing technique (2). For a short time the ablation catheter was placed septally to the line and, during left atrial appendage (LAA) pacing with a circular mapping catheter, the activation sequence on the anterior and septal left atrial (LA) wall was assessed. In the case of a complete block, activation was expected to be septal to lateral. In contrast, a complete block was assumed when, pacing directly and septally to the MAL, the LAA activation occurred later than distal CS activation. If the interval to LAA activation was longer, pacing was performed close to the line. Further evidence of blockages to the line was provided by the presence of double potentials along the MAL during pacing from the LAA.

### 2.3. Late Gadolinium Enhancement Magnetic Resonance Imaging

LGE-MRI was performed during a routine clinical procedure for all patients 3 months after the ablation. LGE-MRI exams were performed on a 3 T Verio clinical MRI (Siemens Medical Solutions, Erlangen, Germany) using a TIM phased-array receiver coil. The scan was acquired in 15 min following contrast agent injection (0.1 mmol/kg, Dotarem (Guerbet)) using a three-dimensional inversion recovery, respiration navigated, electrocardiogram (ECG)-gated, gradient echo pulse sequence. Typical acquisition parameters were: free-breathing using navigator gating, a transverse imaging volume with voxel size  =  1.25 × 1.25 × 2.5 mm (reconstructed to 0.625 × 0.625 × 1.25 mm), Repetition time (TR)/Echo time (TE)  =  5.4/2.3 ms, inversion time (TI)  =  270−310 ms. GRAPPA GeneRalized Autocalibrating Partial Parallel Acquisition) had *R*  =  2 and 46 reference lines. Electrocardiogram gating was used to acquire a small subset of phase encoding views during the diastolic phase of the left atrium (LA) cardiac cycle. The time interval between the *R*-peak of the ECG and the start of data acquisition was defined using the cine images of the LA. Fat saturation was used to suppress fat signals. The TE of the scan (2.3 ms) was chosen such that fat and water were out of phase and the signal intensity of partial volume fat-tissue voxels reduced, allowing improved delineation of the LA wall boundary. The TI value for the LGE-MRI scan was identified using a scout scan. Typical scan time for the LGE-MRI study was 5–10 min depending on subject respiration and heart rate.

To assess the severity of LA late-gadolinium enhancement, post-processing of the LGE-MRI was performed at Merisight Services’ core image-processing center (Merisight, Salt Lake City, UT, USA). The calculation of LA LGE was obtained using LA segmentation and quantification protocols using high-resolution ECG-gated MRA and LGE image stacks, as described previously [12]. In brief, LA wall volumes were carefully obtained through semi-automated and manual segmentation protocols using the subtraction of epicardial and endocardial segmentation, and tailored to exclude the mitral valve and pulmonary veins. The final LA segmentation included the 3D extent of both the LA wall and the pulmonary vein antra. Severity of LA LGE was quantified using a threshold-based algorithm. The number of voxels in the LA wall segmentation with values above the intensity threshold were divided by the total number of voxels in the LA wall segmentation to derive the percentage of LA LGE. LA LGE was categorized based on stages of severity, as described previously.

### 2.4. Analysis of Scar

The MAL was divided into three segments: segment 1 (S1) from mitral annulus to height of lower begin of left atrial appendage (LAA) antrum; segment 2 (S2) height of lower begin of LAA antrum to end of upper LAA antrum; segment 3 (S3) from end of upper LAA antrum to left superior pulmonary vein (Figure 1). The patients were divided into two groups depending on whether or not scar tissue was detectable using LGE-MRI.

### 2.5. Statistical Analysis

All statistical analyses were performed using SPSS (version 23, IBM Corp. Armonk, NY, USA). Continuous variables were presented as median (first quartile to third quartile) and compared using the Mann-Whitney U test. Receiver-operator characteristic (ROC) curves were calculated for setting 1 to set the best threshold and dichotomize the ablation time and the ablation index as a predictor of permanent scar lesion. The area under the curve (AUC) was calculated afterward. A *p*-value of <0.05 was considered statistically significant.

## 3. Results

### 3.1. Acute Results

The MAL was deployed effectively in all patients. The mean AI and CF was 453.9 ± 70.9 and 17.1 ± 5.1 g, respectively. The total number of VisiTag tags was 859 (260 in S1, 277 in S2 and 322 in S3 of MAL), with a mean of 35.8 ± 18.9 per patient for MAL.

In four out of five patients with perimitral flutter, the tachycardia terminated during ablation. In one patient a cardioversion was performed and ablation was continued in sinus rhythm until line of block was confirmed. All patients with persistent AF had a cardioversion. At the end of the procedure, a bidirectional block could be confirmed in all patients. No complications occurred.

### 3.2. LGE-MRI Results

**Figure 2:**
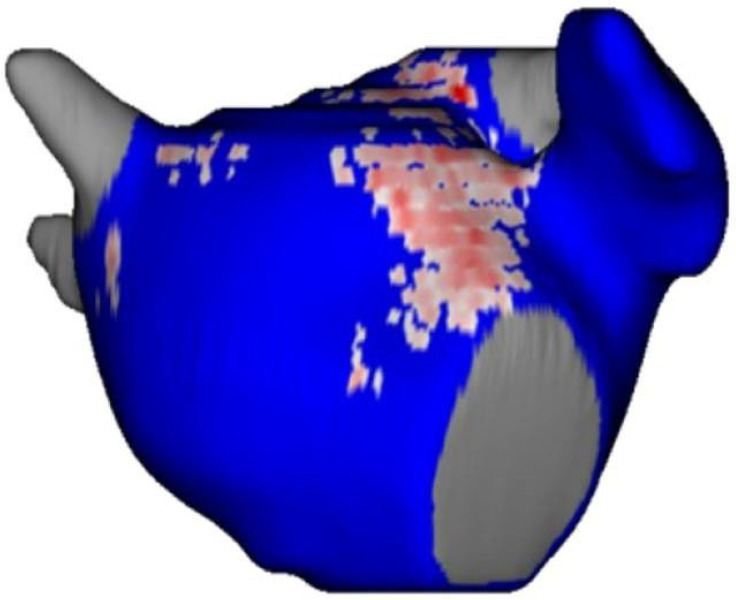
Three-dimensional model of the left atrium. Blue reflects healthy LA tissue, whereas scar areas are shown in red (Merisight TM).

Three months after ablation, LGE-MRI detected durable scars in 45 of 72 segments (Figure 2).

**Figure 3:**
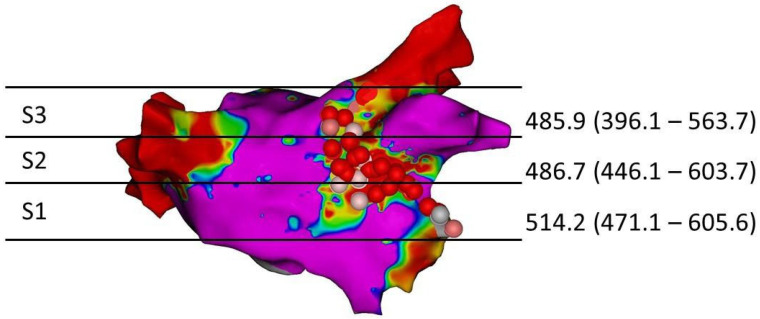
Median AI values for each of the 3 MAL segments for detecting a scar.

The mean AI to create a permanent scar detected using LGE-MRI was 514.2 (471.1–605.6) for S1, 486.7 (446.1–603.7) S2 and 485.9 (396.1–563.7) S3 (Figure 3).

**Figure 4:**
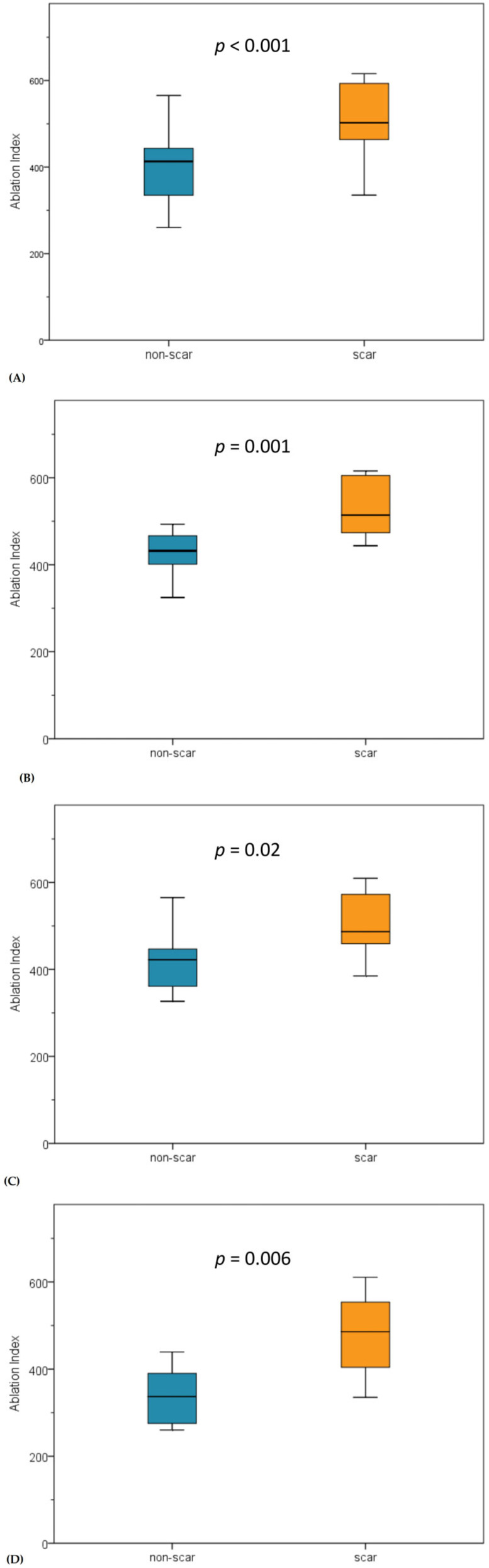
Comparison of AI values. In all segments of the MAL AI values were significantly higher when a scar was identified. (**A**) Total modified anterior line. (**B**) Segment 1 of modified anterior line. (**C**) Segment 2 of modified anterior line. (**D**) Segment 3 of modified anterior line.

The ablation time per VisiTag and AI values were significantly higher when a scar was detected. There was no significant difference in mean CF, which was approximately 17 g in both non-scar and scar groups. Impedance drop was similar between both groups. These results were reproducible when considering each segment of the MAL (Table 2). The boxplots of AI values for total MAL and each segment are depicted in Figure 4.

**Figure 5:**
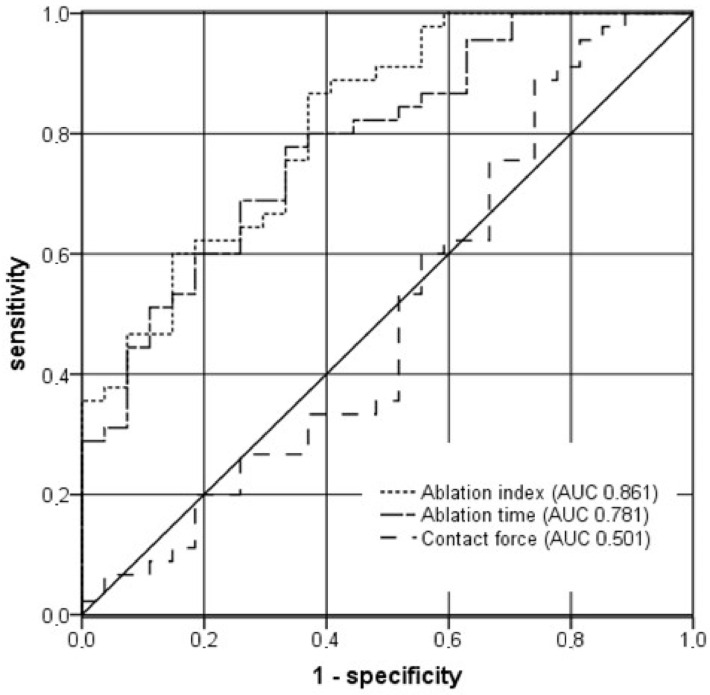
Receiver-operating curves (ROC) for ablation index, ablation time and contact force. AI had the highest accuracy to predict scar (AUC 0.816; *p* < 0.001) with a cut-off value of 421 (sensitivity 0.87; specificity 0.63). Additionally, ablation time showed a high accuracy for predicting scar (AUC 0.784; *p* < 0.001). The point on the ROC curve associated with the greatest discriminatory potential was 14.7 s (sensitivity 0.867; specificity 0.44). CF did not predict scarring, with an AUC of 0.501.

Ablation time and CF to differentiate scar from non-scar was determined using ROC curve analysis, the most discriminant cut-off value for AI (Figure 5).

## 4. Discussion

### 4.1. Main Findings

The main findings of this study are (1) that AI and ablation time were significant discriminators between the emergence and non-emergence of a chronic ablation (the mean CF did not seem to play a significant role) and (2) ROC curves showed that AI and ablation time had a high accuracy in predicting a chronic scar.

RF is effective for PVI. Recent advances in technology, in particular development of ablation index, have shown improvement of ablation outcome [13,14]. Nevertheless, arrhythmia recurrences after catheter ablation are still a clinical reality. Individualized ablation concepts integrating relevant parameters such as the wall thickness could help create durable scar lesions and predict favorable long-term outcomes [15]. As the LA wall has a variable thickness, a general stringent setting would result in over- or undertherapy, leading to a higher risk of complications or ineffective ablation [16,17].

In our study, we retrospectively attempted to find the ablation settings which predict a durable scar on MAL. We found no significant difference between the mean CF in scar and non-scar areas. It was of note that ablation lesions with a CF of less than 3 g were not tagged. Previous studies reported that CF could be well-used to mark good catheter tissue contact in atrial ablation. Importantly, in post-ablation LGE-MRI, there was no difference in ablation lesion size whether the force was 8 or 23 g [18,19]. It could also be shown that lesions could be created, even with minimal force, as long as there is good contact [20]. Accordingly, in our study the CF magnitude appeared not to play a main role in predicting a chronic scar provided good contact was established. Incidentally, we regularly used a steerable sheath, enabling better and more continuous tissue contact than with a non-steerable one. This might have precluded measuring the impact of contact force. However, these clinical data are not in line with in vitro studies, showing a strong correlation of contact force and lesion size [17,21]. This could be explained by limited resolution of LGE-MRI or by other factors influencing ablation lesion and reducing accuracy of force measurements, such as catheter orientation and electrode coverage [22]. Of note, impedance decrease during RF application, which is known as a reliable parameter of lesion formation [14,23], was not an independent predictor of chronic scarring in our study. Thus, we could show that only ablation time and, as a result, the AI were the most relevant parameters in predicting a durable scar. This finding could easily be translated to clinical practice.

Importantly, our results could only be assigned to a conventional low-power (30–40 W), long duration (20–30 s.) approach. Recently, high-power, short-duration (HPSD) ablation for AF treatment emerged as an alternative. The potential advantages of HPSD ablation include less tissue oedema and collateral tissue damage, a reduction in procedural time and improved lesion-to-lesion uniformity, linear contiguity and transmurality [24,25,26]). At present, there is no consensus in definition for what constitutes HPSD ablation, which varied from 50 to 90 W. This variation is also reflected in lesion geometry [27]. Even if the focus of the recent study from Zanchi et al. centred on acute ablation success, and the difference of the power setting was lower compared with our study, they could still show that AI-guided high-power (50 W) ablation, targeting AI 500, seems to be a feasible technique with high first pass block rate for anterior line ablation [28]. However, AI is not validated for HPSD and more clinical data are required for line ablation with HPSD, especially with regard to long-term results.

In our study, we used the LGE-MRI to assess lesion formation. This technique was recently introduced as a non-invasive method to visualize the effects of the ablation procedure [12]. Badger et al. demonstrated that LGE-MRI can accurately define scar lesions after AF ablation. [29]. In this earlier study, all patients demonstrated similar distribution in the extent and location of low voltage tissue on electroanatomical maps and scar tissue on LGE-MRI. Recent studies show that the formation of novel fibrosis post-ablation and subsequent LGE-MRI could provide accurate guidance for redo-procedures and might act as a marker for long-term outcomes [30,31]. Nonetheless, larger studies would have to evaluate the predicted value and the clinical impact of detecting scar tissue using LGE-MRI.

### 4.2. Limitation

First, this study was a single center study including a relatively small group of patients. However, almost 900 ablation tags were assessed, which allowed us to perform a robust statistical analysis. Second, important parameters influencing ablation lesion, such as wall thickness along the line, could not be measured. Third, we did not perform an electrophysiological study in all patients to provide a correlation between scar and line of block. Finally, LGE-MRI has a number of imitating factors that can affect imaging quality and its significance. However, using standardized protocols, several authors could reproduce similar results after PVI, showing a correlation between post ablation scar tissue detected by LGE-MRI and low voltage areas in anatomical mapping [29,30]. Our study is the first evaluating this technique in left atrial linear lesions.

## 5. Conclusions

Targeting optimal AI values is crucial in determining left atrial scar tissue using LGE-MRI 3 months after ablation. AI-guided linear left atrial ablation seems to be effective at producing durable lesions.

## Figures and Tables

**Table 1:** Baseline characteristics. (Plus-minus values are means ± SD; LA, left atrium; LVEF, left. ventricular ejection fraction).

Patient Characteristics (n = 24)	
Age, years	72 ± 9.3
Male, %	45.8
CHA2DSVASc Score	3.1 ± 1.1
Body mass index, kg/m^2^	30.5 ± 5.9
LA-diameter, mm	51.9 ± 7.2
LVEF, %	57.1 ± 12.4
Time of LGE-CMR after ablation, days	97.5 ± 14.6

**Table 2:** Ablation parameters compared non-scar vs. scar (continuous variables presented as median (first to third quartile) and compared using Mann-Whitney U Test).

	Non-Scar	Scar	*p* Value
**Total MAL**			
N	295	564	
AI	413.19 (326.84–446.97)	502.1 (450.2–583.4)	<0.0001
Impedance drop, ohms	4.8 (3.4–6.3)	5.6 (3.8–6.8)	0.118
Force, g	17.9 (11.9–19.9)	17.1 (13.9–19.3)	0.986
Time, s	15.5 (12.2–18.8)	19.5 (16.6–26.3)	<0.0001
S1			
N	88	172	
AI	432.1 (395.3–474.8)	514.2 (471.1–605.6)	0.001
Impedance drop, ohms	4.5 (3.1–6.6)	5.7 (3.7–6.7)	0.391
Force, g	18.5 (14.8–26.9)	17.6 (13.7–21.2)	0.668
Time, s	15.8 (13.2–17.5)	20.4 (17.8–23.9)	0.012
S2			
N	92	185	
AI	422.4 (352.1–481.6)	486.7 (446.1–603.7)	0.020
Impedance drop, ohms	5.2 (4.0–6.9)	5.6 (4.0–6.5)	1.000
Force, g	18.1 (15.1–19.4)	15.6 (13.8–19.6)	0.519
Time, s	15.4 (12.1–19.1)	22.1 (17.8–28.7)	0.018
S3			
N	115	207	
AI	336.8 (272.2–402.5)	485.9 (396.1–563.7)	0.006
Impedance drop, ohms	3.9 (3.2–5.7)	6.1 (4.5–8.9)	0.089
Force, g	13.8 (9.6–19.4)	16.8 (14.1–18.1)	0.482
Time, s	13.9 (9.1–19.1)	20.2 (14.6–27.7)	0.026

AI, ablation index; MAL, modified anterior line; N, number of ablation tags; S, segment of MAL).

## Data Availability

The data presented in this study are available on request from the corresponding author.

## References

[1] Matsuo S., Wright M., Knecht S., Nault I., Lellouche N., Lim K.T., Arantes L., O’Neill M.D., Hocini M., Jaïs P. (2010). Peri-mitral atrial flutter in patients with atrial fibrillation ablation. Heart Rhythm.

[2] Ammar S., Luik A., Hessling G., Bruhm A., Reents T., Semmler V., Buiatti A., Kathan S., Hofmann M., Kolb C. (2015). Ablation of perimitral flutter: Acute and long-term success of the modified anterior line. Europace.

[3] Kircher S., Arya A., Altmann D., Rolf S., Bollmann A., Sommer P., Dagres N., Richter S., Breithardt O.A., Dinov B. (2018). Individually tailored vs. standardized substrate modification during radiofrequency catheter ablation for atrial fibrillation: A randomized study. Europace.

[4] Tzeis S., Luik A., Jilek C., Schmitt C., Estner H.L., Wu J., Reents T., Fichtner S., Kolb C., Karch M.R. (2010). The modified anterior line: An alternative linear lesion in perimitral flutter. J. Cardiovasc. Electrophysiol..

[5] Calkins H., Hindricks G., Cappato R., Kim Y.H., Saad E.B., Aguinaga L., Akar J.G., Badhwar V., Brugada J., Camm J. (2018). 2017 HRS/EHRA/ECAS/APHRS/SOLAECE expert consensus statement on catheter and surgical ablation of atrial fibrillation. Europace.

[6] Jais P., Haissaguerre M., Shah D.C., Takahashi A., Hocini M., Lavergne T., Lafitte S., Le Mouroux A., Fischer B., Clementy J. (1998). Successful irrigated-tip catheter ablation of atrial flutter resistant to conventional radiofrequency ablation. Circulation.

[7] Haines D.E., Strunk A.R., Novichenok A., Kirchhof N., Stewart M. (2015). The Biophysics of Passive Convective Cooling During Catheter Ablation with Gold versus Platinum Electrodes and Multielectrode Phased Radiofrequency Energy Delivery. J. Cardiovasc. Electrophysiol..

[8] Bisbal F., Guiu E., Cabanas-Grandio P., Berruezo A., Prat-Gonzalez S., Vidal B., Garrido C., Andreu D., Fernandez-Armenta J., Tolosana J.M. (2014). CMR-guided approach to localize and ablate gaps in repeat AF ablation procedure. JACC Cardiovasc. Imaging.

[9] Chubb H., Aziz S., Karim R., Sohns C., Razeghi O., Williams S.E., Whitaker J., Harrison J., Chiribiri A., Schaeffter T. (2018). Optimization of late gadolinium enhancement cardiovascular magnetic resonance imaging of post-ablation atrial scar: A cross-over study. J. Cardiovasc. Magn. Reson. Off. J. Soc. Cardiovasc. Magn. Reson..

[10] Spragg D.D., Khurram I., Zimmerman S.L., Yarmohammadi H., Barcelon B., Needleman M., Edwards D., Marine J.E., Calkins H., Nazarian S. (2012). Initial experience with magnetic resonance imaging of atrial scar and co-registration with electroanatomic voltage mapping during atrial fibrillation: Success and limitations. Heart Rhythm.

[11] Yamashita K., Kamali R., Kwan E., MacLeod R.S., Dosdall D.J., Ranjan R. (2020). Effective Ablation Settings That Predict Chronic Scar After Left Atrial Ablation. JACC Clin. Electrophysiol..

[12] Akoum N., Wilber D., Hindricks G., Jais P., Cates J., Marchlinski F., Kholmovski E., Burgon N., Hu N., Mont L. (2015). MRI Assessment of Ablation-Induced Scarring in Atrial Fibrillation: Analysis from the DECAAF Study. J. Cardiovasc. Electrophysiol..

[13] Phlips T., Taghji P., El Haddad M., Wolf M., Knecht S., Vandekerckhove Y., Tavernier R., Duytschaever M. (2018). Improving procedural and one-year outcome after contact force-guided pulmonary vein isolation: The role of interlesion distance, ablation index, and contact force variability in the ‘CLOSE’-protocol. Europace.

[14] Hussein A., Das M., Riva S., Morgan M., Ronayne C., Sahni A., Shaw M., Todd D., Hall M., Modi S. (2018). Use of Ablation Index-Guided Ablation Results in High Rates of Durable Pulmonary Vein Isolation and Freedom From Arrhythmia in Persistent Atrial Fibrillation Patients: The PRAISE Study Results. Circ. Arrhythmia Electrophysiol..

[15] Teres C., Soto-Iglesias D., Penela D., Jauregui B., Ordonez A., Chauca A., Huguet M., Ramirez-Paesano C., Oller G., Jornet A. (2021). Left atrial wall thickness of the pulmonary vein reconnection sites during atrial fibrillation redo procedures. Pacing Clin. Electrophysiol. PACE.

[16] Beinart R., Abbara S., Blum A., Ferencik M., Heist K., Ruskin J., Mansour M. (2011). Left atrial wall thickness variability measured by CT scans in patients undergoing pulmonary vein isolation. J. Cardiovasc. Electrophysiol..

[17] Ikeda A., Nakagawa H., Lambert H., Shah D.C., Fonck E., Yulzari A., Sharma T., Pitha J.V., Lazzara R., Jackman W.M. (2014). Relationship between catheter contact force and radiofrequency lesion size and incidence of steam pop in the beating canine heart: Electrogram amplitude, impedance, and electrode temperature are poor predictors of electrode-tissue contact force and lesion size. Circ. Arrhythmia Electrophysiol..

[18] Williams S.E., Harrison J., Chubb H., Bloch L.O., Andersen N.P., Dam H., Karim R., Whitaker J., Gill J., Cooklin M. (2015). The Effect of Contact Force in Atrial Radiofrequency Ablation: Electroanatomical, Cardiovascular Magnetic Resonance, and Histological Assessment in a Chronic Porcine Model. JACC Clin. Electrophysiol..

[19] Andreu D., Gomez-Pulido F., Calvo M., Carlosena-Remirez A., Bisbal F., Borras R., Benito E., Guasch E., Prat-Gonzalez S., Perea R.J. (2016). Contact force threshold for permanent lesion formation in atrial fibrillation ablation: A cardiac magnetic resonance-based study to detect ablation gaps. Heart Rhythm.

[20] Thomas S., Silvernagel J., Angel N., Kholmovski E., Ghafoori E., Hu N., Ashton J., Dosdall D.J., MacLeod R., Ranjan R. (2018). Higher contact force during radiofrequency ablation leads to a much larger increase in edema as compared to chronic lesion size. J. Cardiovasc. Electrophysiol..

[21] Yokoyama K., Nakagawa H., Shah D.C., Lambert H., Leo G., Aeby N., Ikeda A., Pitha J.V., Sharma T., Lazzara R. (2008). Novel contact force sensor incorporated in irrigated radiofrequency ablation catheter predicts lesion size and incidence of steam pop and thrombus. Circ. Arrhythmia Electrophysiol..

[22] Bourier F., Popa M., Kottmaier M., Maurer S., Bahlke F., Telishevska M., Lengauer S., Koch-Buttner K., Kornmayer M., Risse E. (2021). RF electrode-tissue coverage significantly influences steam pop incidence and lesion size. J. Cardiovasc. Electrophysiol..

[23] Chinitz J.S., Kapur S., Barbhaiya C., Kumar S., John R., Epstein L.M., Tedrow U., Stevenson W.G., Michaud G.F. (2016). Sites With Small Impedance Decrease During Catheter Ablation for Atrial Fibrillation Are Associated With Recovery of Pulmonary Vein Conduction. J. Cardiovasc. Electrophysiol..

[24] Leshem E., Zilberman I., Tschabrunn C.M., Barkagan M., Contreras-Valdes F.M., Govari A., Anter E. (2018). High-Power and Short-Duration Ablation for Pulmonary Vein Isolation: Biophysical Characterization. JACC Clin. Electrophysiol..

[25] Ali-Ahmed F., Goyal V., Patel M., Orelaru F., Haines D.E., Wong W.S. (2019). High-power, low-flow, short-ablation duration-the key to avoid collateral injury?. J. Interv. Card. Electrophysiol. Int. J. Arrhythm. Pacing.

[26] Winkle R.A., Mohanty S., Patrawala R.A., Mead R.H., Kong M.H., Engel G., Salcedo J., Trivedi C.G., Gianni C., Jais P. (2019). Low complication rates using high power (45-50 W) for short duration for atrial fibrillation ablations. Heart Rhythm.

[27] Bourier F., Duchateau J., Vlachos K., Lam A., Martin C.A., Takigawa M., Kitamura T., Frontera A., Cheniti G., Pambrun T. (2018). High-power short-duration versus standard radiofrequency ablation: Insights on lesion metrics. J. Cardiovasc. Electrophysiol..

[28] Zanchi S., Chen S., Bordignon S., Bianchini L., Tohoku S., Bologna F., Tondo C., Chun K.R.J., Schmidt B. (2021). Ablation Index-guided high-power (50 W) short-duration for left atrial anterior and roofline ablation: Feasibility, procedural data, and lesion analysis (AI High-Power Linear Ablation). J. Cardiovasc. Electrophysiol..

[29] Badger T.J., Daccarett M., Akoum N.W., Adjei-Poku Y.A., Burgon N.S., Haslam T.S., Kalvaitis S., Kuppahally S., Vergara G., McMullen L. (2010). Evaluation of left atrial lesions after initial and repeat atrial fibrillation ablation: Lessons learned from delayed-enhancement MRI in repeat ablation procedures. Circ. Arrhythmia Electrophysiol..

[30] Althoff T.F., Garre P., Caixal G., Perea R., Prat S., Tolosana J.M., Guasch E., Roca-Luque I., Arbelo E., Sitges M. (2022). Late gadolinium enhancement-MRI determines definite lesion formation most accurately at 3 months post ablation compared to later time points. Pacing Clin. Electrophysiol. PACE.

[31] Kheirkhahan M., Baher A., Goldooz M., Kholmovski E.G., Morris A.K., Csecs I., Chelu M.G., Wilson B.D., Marrouche N.F. (2020). Left atrial fibrosis progression detected by LGE-MRI after ablation of atrial fibrillation. Pacing Clin. Electrophysiol. PACE.

